# Morphological, transcriptomic and metabolomic analyses of *Sophora davidii* mutants for plant height

**DOI:** 10.1186/s12870-022-03503-1

**Published:** 2022-03-25

**Authors:** Xin Zhao, Xiao-Fu Sun, Li-Li Zhao, Li-Juan Huang, Pu-Chang Wang

**Affiliations:** 1 College of Animal Science, Guizhou University, Guiyang, 550025 China; 2 Weining Plateau Grassland Test Station, Weining, 553100 China; 3 Guizhou Institute of Prataculture, Guiyang, 550006 China

**Keywords:** *Sophora davidii* mutants, Plant height, Phenotypic, Transcriptomics, Metabolomics

## Abstract

*Sophora davidii* is an important plant resource in the karst region of Southwest China, but *S. davidii* plant-height mutants are rarely reported. Therefore, we performed phenotypic, anatomic structural, transcriptomic and metabolomic analyses to study the mechanisms responsible for *S. davidii* plant-height mutants. Phenotypic and anatomical observations showed that compared to the wild type, the dwarf mutant displayed a significant decrease in plant height, while the tall mutant displayed a significant increase in plant height. The dwarf mutant cells were smaller and more densely arranged, while those of the wild type and the tall mutant were larger and loosely arranged. Transcriptomic analysis revealed that differentially expressed genes (DEGs) involved in cell wall biosynthesis, expansion, phytohormone biosynthesis, signal transduction pathways, flavonoid biosynthesis and phenylpropanoid biosynthesis were significantly enriched in the *S. davidii* plant-height mutants. Metabolomic analysis revealed 57 significantly differential metabolites screened from both the dwarf and tall mutants. A total of 8 significantly different flavonoid compounds were annotated to LIPID MAPS, and three metabolites (chlorogenic acid, kaempferol and scopoletin) were involved in phenylpropanoid biosynthesis and flavonoid biosynthesis. These results shed light on the molecular mechanisms of plant height in *S. davidii* mutants and provide insight for further molecular breeding programs.

**Supplementary Information:**

The online version contains supplementary material available at 10.1186/s12870-022-03503-1.

## Introduction

Plant height is an important component of plant structure and an important agronomic trait that affects crop yields and plant quality. Changes in plant height are one of the most obvious features of the growth and development process. Plant dwarf breeding is extremely important in production [1]. Dwarf plants can show an improved plant shapes, increases in planting density and plant population photosynthetic efficiency, reduced pruning requirements, low water consumption, lodging resistance, and enhanced transportation of crop nutrients to reproductive organs. Plant dwarfing can also reduce the use of chemical growth regulators (such as dwarfing agents), which is conducive to protecting the ecological environment [2]. However, studies have also shown that increasing plant heights can increase plant biological yields, which is conducive to the mechanical harvesting of low-rise plants and increases the recovery rate [3]. The mining and study of genes related to the ideal plant height in higher plants have long been a focus of research in the life sciences [4, 5]. Therefore, researchers select plant varieties with height-related mutations as research targets, as they serve as good materials for studying plant height regulation, plant hormone biosynthesis, signal transduction, and stem growth and development.

In addition to external environmental influences on plant height, the expression and regulation of genes related to plant height and growth play dominant roles in plants. Gibberellin (GA), brassinosteroid (BR), and auxin (IAA) hormones have important effects on plant height. In addition, these hormones also interact with ethylene, jasmonic acid and stratolactone during trait development [6]. To date, 7 genes related to GA synthesis have been cloned in rice, including *sd1*, *d35*, *d18*, *OsKS1*, *eui1*, *OsGA2ox6* and *OsGA13ox*. The sd1 mutant exhibits a semidwarfing phenotype due to the mutation of *GA20ox* oxidase in rice, which hinders the oxidation of *GA53* to *GA20* [7]. Han et al. [8] found that the overexpression of the BR degradation-related gene *AtBAT1* in transgenic creeping bentgrass resulted in plant dwarfing, shorter internodes, smaller leaf angles and other BR deficiency-related phenotypes. The abnormal expression of key auxin transport genes can cause plant dwarfing. In *Arabidopsis* pin, abcb, mdr, pgp, and maize br2 mutants, the dwarfing phenotype of plants appears due to the lack or obstruction of auxin transport function [9, 10]. Campanoni and Nick [11] found that auxin-binding protein 1 (*ABP1*) induces the division of tobacco cells and promotes the elongation of tobacco stems.

In addition to hormonal pathways, many nonhormonal pathways in plants are involved in high-level plant regulation, including cell wall development pathways, cytoplasmic glutamine synthesis pathways, cell division, ubiquitin-protease degradation pathways, and fatty acid metabolism pathways. Mutations in important genes in these pathways are usually accompanied by symptoms such as plant dwarfing, leaf curling, and reduced fertility [12]. The OsCD1 gene in rice encodes a member of the D subfamily of cellulose synthases, which is involved in cell wall formation. cd1 mutants have significantly reduced cellulose and xylose contents in stalks and show dwarfism, curled and narrow leaves and reduced grain numbers in spikes. cd1 mutants respond to GA3 in the same way as wild-type rice, suggesting that this gene regulates plant height development by a mode independent of plant hormones [13]. Guo et al. [14] found that genes including CesA and CSL are responsible for cell wall biogenesis, along with expansins and *XTH*, which are involved in cell wall loosening and are critical to plant growth and development. Tanaka et al. identified three cellulose synthase catalytic subunit (*CesA*) genes, *OsCesA4*, *OsCesA7*, and *OsCesA9*. Mutants of these genes showed semidwarfing, fragile stalks, and reduced fertility, indicating that these three genes are functionally redundant; thus, they may encode protein complexes involved in cell wall synthesis [15].

*S davidii*, which belongs to a subfamily of shrubs, is a perennial shrub that showns drought tolerance, barren resistance and trample resistance. It has deep roots, a strong sprouting ability and the capacity for biological nitrogen fixation. It is a pioneer tree species used for conservation, vegetation restoration, soil improvement and afforestation. It is also a high-quality feed source whose leaves and stems are tender, palatable and rich in protein, vitamins, mineral elements and amino acids. Additionally, the seeds, flowers, leaves and roots of *S. davidii* contain a variety of alkaloids, flavonoids and other medicinal components with various pharmacological activities, such as antitumor, antibacterial, anti-inflammatory, antiallergic, antiviral and antioxidative activities [16–18]. However, studies on *S. davidii* plant-height mutants are rarely reported. In previous research, we obtained plant-height mutants by using 60Co-γ radiation [19, 20]. In this study, we therefore aimed to reveal differentially expressed genes (DEGs) and their associated metabolic pathways in these height mutants by transcriptome and metabolome sequencing and to subsequently present possible reasons for the resulting pleiotropic effects. We analyzed and discussed the mechanisms responsible for the *S. davidii* plant-height mutants based on morphological variation, anatomical structure, endogenous hormone content and the relationships between DEGs and different plant-height mutants. Moreover, quantitative reverse-transcription–polymerase chain reaction (qRT–PCR) analysis verified DEGs involved in hormone signaling, and IAA, BR and GA3 treatments were performed to demonstrate that the expression of these genes was indeed changed. These results shed light on the different molecular mechanisms producing differences between the plant-height mutants and the wild type and provide an effective theoretical basis for *S. davidii* breeding.

## Results

### Distinct phenotypes of the *S. davidii* dwarf mutant, wild type and tall mutant

**Fig. 1:**
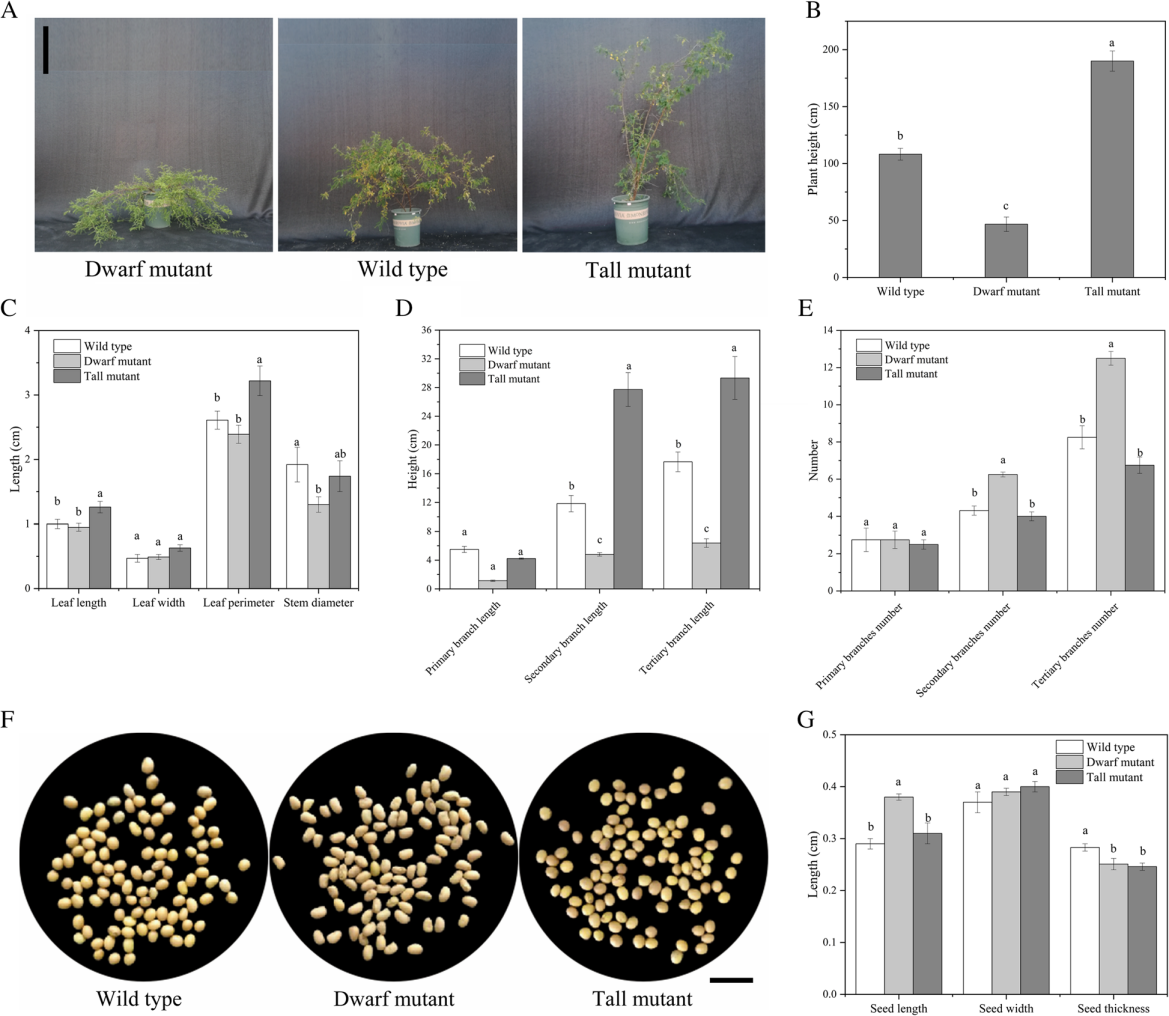
Comparison of phenotypic characteristics among the *S. davidii* dwarf mutant, wild type and tall mutant*.* **A, B** Plant heights of the *S. davidii* dwarf mutant, wild type and tall mutant. Scale bars = 0.5 m **C** Leaf morphology and stem diameters of the *S. davidii* dwarf mutant, wild type and tall mutant. **D** Branch lengths of the *S. davidii* dwarf mutant, wild type and tall mutant. **E** Branch numbers of the *S. davidii* dwarf mutant, wild type and tall mutant*.* **F, G** Seed phenotypes of the *S. davidii* dwarf mutant, wild type and tall mutant. Scale bars = 1 cm. The error bars show the standard error of the mean (SEM). Different letters associated with the same trait indicate significant differences between treatments (*P* < 0.05)

According to the phenotypic data, relative to the wild type, the dwarf mutant displayed significant decreases in plant height (Fig. 1A, B; *P* < 0.05), stem diameter (Fig. 1C; *P* < 0.05), secondary branch length and tertiary branch length (Fig. 1D; *P* < 0.05), and the tall mutant displayed significant increases in plant height (Fig. 1A, B; *P* < 0.05), secondary branch length and tertiary branch length (Fig. 1D; *P* < 0.05). In general, the plant height, primary branch length, secondary branch length and tertiary branch length of the wild type were ˃twofold higher than those of the dwarf mutant. In addition, the tall mutant displayed significant increases in leaf length and leaf perimeter (Fig. 1C; *P* < 0.05). Interestingly, opposite branch number and leaf shape change trends were observed, and the dwarf mutant displayed a significantly increased secondary branch number and tertiary branch number (Fig. 1E; *P* < 0.05). To explore the seed morphological variation in dwarf *S. davidii*, we observed the appearance of the seeds and measured seed morphology. We found that relative to the wild type, the dwarf mutant displayed a significantly increased seed length, and both the dwarf mutant and the tall mutant displayed a significantly decreased seed thickness (Fig. 1G; *P* < 0.05). In contrast to the wild type, the dwarf mutant seeds were yellow or light yellow and wrinkled, while the tall mutant seeds were brown, yellowish brown or yellow and had smooth surfaces (Fig. 1F).

**Fig. 2:**
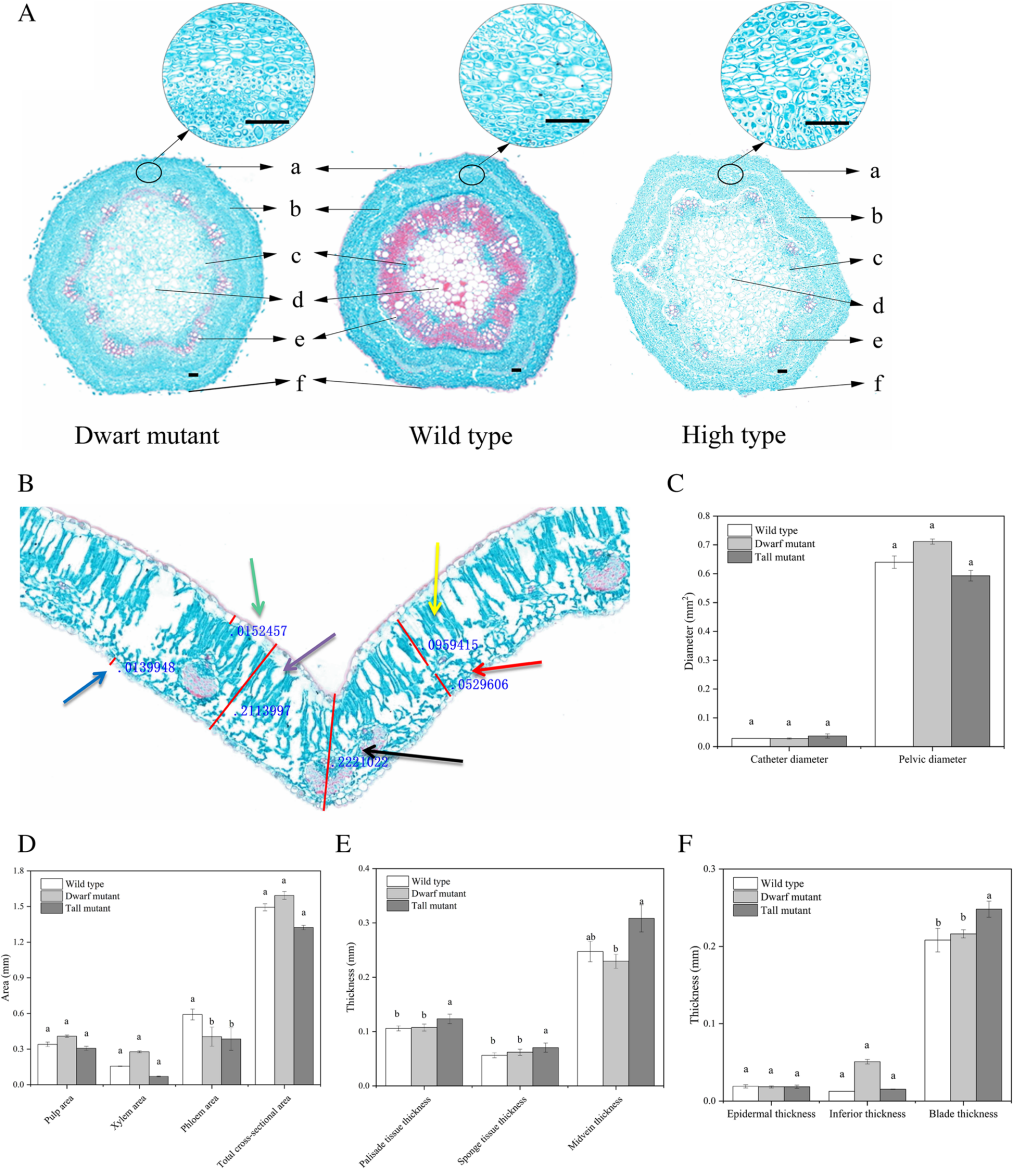
Histological differences among the *S. davidii* dwarf mutant, wild type and tall mutant*.* **A** Fields of view of stem sections of the *S. davidii* dwarf mutant, wild type and tall mutant, a: epidermis; b: phloem; c: xylem; d: catheter; e: medulla; f: total cross-sectional area. All scale bars = 50 μm **B** Example diagram of leaf section index analysis of *S. davidii*, showing the thicknesses of blade (purple arrow), epidermal (green arrow), inferior (blue arrow), midvein (black arrow), palisade (yellow arrow), and sponge (red arrow) tissues. **C, D** Section analysis results of stem sections of the *S. davidii* dwarf mutant, wild type and tall mutant. **E, F** Results of leaf section analysis of the *S. davidii* dwarf mutant, wild type and tall mutant. The error bars show the SEM. Different letters associated with the same trait indicate significant differences between treatments (*P* < 0.05)

Photographs of microscopic observations of the prepared stem segment paraffin sections and a selection of different fields of view under the same magnification are presented herein. The cells of the dwarf mutant were smaller and arranged more closely, while those of the wild type and tall mutant were larger and arranged loosely (Fig. 2A). According to the analysis of stem sections of the dwarf mutant, wild type and tall mutant with Image-Pro Plus 6.0 software, the dwarf mutant and tall mutant displayed significantly decreased phloem areas relative to the wild type (Fig. 2D; *P* < 0.05), whereas no differences in phloem area were detected between the *S. davidii* dwarf mutant and tall mutant*.* No differences in catheter diameter, pelvic diameter, pulp area, xylem area or total cross-sectional area were detected among the *S. davidii* dwarf mutant, wild type and tall mutant (Fig. 2C, D). Image-Pro Plus 6.0 software was used to analyze leaf sections of the *S. davidii* dwarf mutant, wild type and tall mutant (Fig. 2B). Relative to the wild type, the tall mutant displayed significantly increased thicknesses of blade, palisade and sponge tissue, and relative to the tall mutant, the dwarf mutant presented significantly decreased thicknesses of blade, midvein and palisade tissue (Fig. 2E, F; *P* < 0.05). No differences in epidermal or inferior thickness were detected among the *S. davidii* dwarf mutant, wild type and tall mutant (Fig. 2F).

2.2 Global changes in candidate gene expression during shoot apex and lateral branch bud development in the *S. davidii* dwarf mutant, wild type and tall mutant.

To understand the possible mechanisms responsible for *S. davidii* plant-height mutants at the transcriptional level, we sought to identify key candidate genes that influence plant height. We compared the transcriptomic profiles of mature-stage shoots from the *S. davidii* dwarf mutant, wild type and tall mutant. A total of 537 million raw read pairs were generated, and 532 million clean reads were obtained after removing adaptors, low-quality sequences, and contaminant reads (Table 1, the data were deposited into the NCBI Sequence Read Archive (SRA) with the accession: PRJNA783425). The clean rates and the Q20 and Q30 values of the samples reached 97% and 93%, respectively, and approximately 74.06–75.73% of the clean reads were mapped to the reference genome of *S. davidii*, indicating the high quality of the sequencing results.

**Table 1:** Summary of RNA-seq data from the *S. davidii* dwarf mutant, wild type and tall mutant

Sample name	Raw reads	Clean reads	Total mapped (bp)	Error (%)	Q20 (%)	Q30 (%)	Genome map rate (%)
D1	43,765,422	43,307,472	32,075,138	0.03	97.28	92.69	74.06
D2	63,906,450	63,317,948	47,953,280	0.03	97.79	93.73	75.73
D3	45,743,058	45,233,064	33,760,464	0.03	97.67	93.45	74.64
W1	50,391,932	49,829,394	37,012,394	0.03	97.39	92.85	74.28
W2	66,836,592	66,299,494	49,810,428	0.03	97.7	93.54	75.13
W3	64,316,530	63,800,432	47,517,308	0.03	97.75	93.67	74.48
H1	66,793,170	66,081,064	48,984,538	0.03	97.61	93.44	74.13
H2	64,516,072	64,016,786	48,437,926	0.03	97.82	93.8	75.66
H3	70,818,898	70,240,948	52,898,342	0.03	97.64	93.37	75.31

2.3 DEGs involved in cell wall biosynthesis and expansion

We identified the DEGs in the *S. davidii* dwarf mutant, wild type and tall mutant. A total of 2175 DEGs were found between the *S. davidii* dwarf vs. wild type, 2570 DEGs were found between the *S. davidii* wild type and tall mutant, and 2602 were found between the *S. davidii* dwarf mutant and the tall mutant (Supplemental Table S1). These differences could exist because in mature *S. davidii*, most functional cells are already relatively mature, and the large number of DEGs represents genes that are differentially transcribed to maintain the morphological differences among the *S. davidii* dwarf mutant, wild type and tall mutant.

Gene Ontology (GO) enrichment analyses revealed that in the *S. davidii* dwarf mutant, wild type and tall mutant, cell division and anatomical structure morphogenesis-related genes showed significant differences in expression levels. In the shoot apexes or lateral branch buds, cell wall biosynthesis- and expansion-related genes were the most significantly upregulated DEGs in the *S. davidii* dwarf mutant, wild type and tall mutant (Supplemental Table S2).

**Fig. 3:**
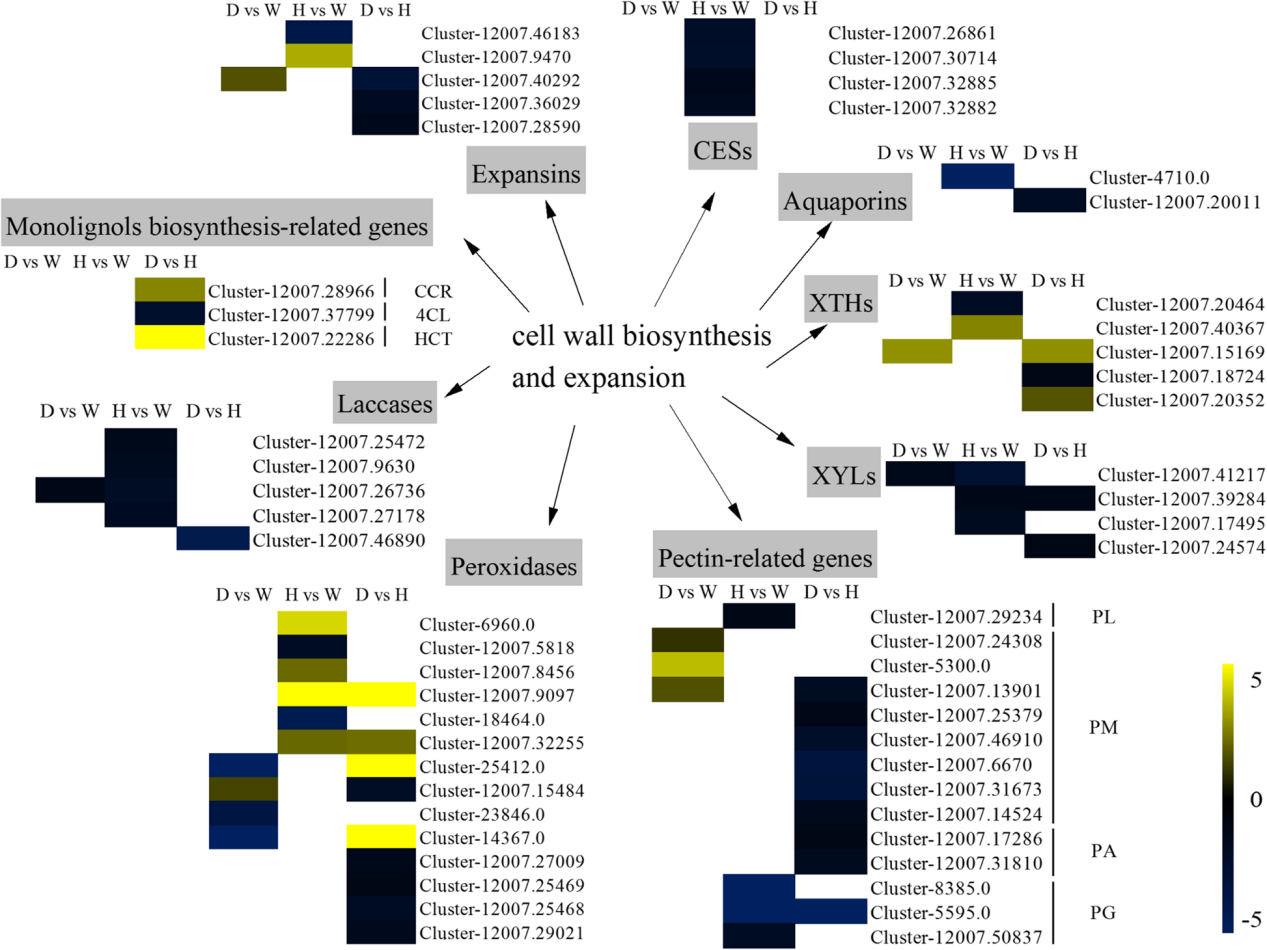
Heatmaps showing the relative changes in lignin biogenesis- and fiber expansion-related gene expression in the *S. davidii* dwarf mutant, wild type and tall mutant. Yellow and blue indicate up- and downregulated transcripts, respectively, identified in the three comparisons, and white indicates no significant difference in expression. 4CL: 4-coumarate-CoA ligase; CCR: cinnamoyl-CoA reductase; HCT: p-hydroxycinnamoyl-CoA: quinate/shikimate O-hydroxycinnamoyltransferase. XTH: xyloglucan endotransglucosylase/hydrolase; CES: cellulose synthase; XYL: xylosidase; PL: pectate lyase; PM: pectinesterase; PA: pectin acetylesterase; PG: polygalacturonase. All genes are listed in Supplemental Table S3

A comparative expression analysis was performed for the 4-coumarate-CoA ligase (*4CL*), cinnamoyl-CoA reductase (*CCR*), and p-hydroxycinnamoyl-CoA: quinate/shikimate O-hydroxycinnamoyltransferase (*HCT*) genes, which are involved in the biosynthesis of the three types of monolignols (p-coumaryl, sinapyl, and coniferyl alcohols) and are thought to play critical roles in regulating lignin accumulation in plants [21]. In this study, the *CCR* and *HCT* genes were significantly upregulated and the *4CL* gene was significantly downregulated in the dwarf vs. tall groups of *S. davidii*. The expression of peroxidase and laccase genes, which participate in the oxidation and polymerization of monolignols and in the final step in the formation of lignin polymers, was also examined. Laccase genes were significantly downregulated in the *S. davidii* dwarf mutant, wild type and tall mutant. Three peroxidase genes were significantly downregulated and one peroxidase gene was significantly upregulated in the dwarf vs. wild-type groups; four peroxidase genes were significantly upregulated and two were downregulated in the tall mutant relative to the wild type; and four peroxidase genes were significantly upregulated and five were downregulated in the dwarf vs. tall groups (Fig. 3 and Supplemental Table S3).

All DEGs related to cellulose biosynthesis, which encoded cellulose synthase A catalytic subunits (*CESAs*), were downregulated in the tall mutant relative to wild-type *S. davidii*. Loosening of the plant cell wall is a necessary physiological process that occurs during cell expansion and elongation throughout the entire period of growth and development in plants [22]. Expansins (EXPs) are nonhydrolytic cell wall-loosening proteins that enable cells to expand while allowing tissues to differentiate and grow [23]. Five putative members of the *EXP* gene family were differentially expressed in *S. davidii*; one *EXP* was significantly upregulated in the dwarf vs. wild-type groups; one *EXP* was significantly upregulated and one was downregulated in the tall vs. wild-type groups; and three *EXP*s were significantly downregulated in the dwarf vs. tall groups. Xyloglucan endotransglucosylase/hydrolase (*XTH*) has also been shown to be involved in cell expansion by loosening and rearranging the cell wall fibers in growing tissues. One *XTH* was significantly upregulated in the dwarf vs. wild-type groups; one *XTH* was significantly upregulated and one was downregulated in the tall vs. wild-type groups; and two XTHs were significantly upregulated and one was downregulated in the dwarf vs. tall groups. Moreover, the relative expression levels of one *PL*, eight *PM*, two *PA*, four *XYL*, three *PG*, and two aquaporin genes showed significant differences.

### DEGs involved in phytohormone biosynthesis and signal transduction pathways

**Fig. 4:**
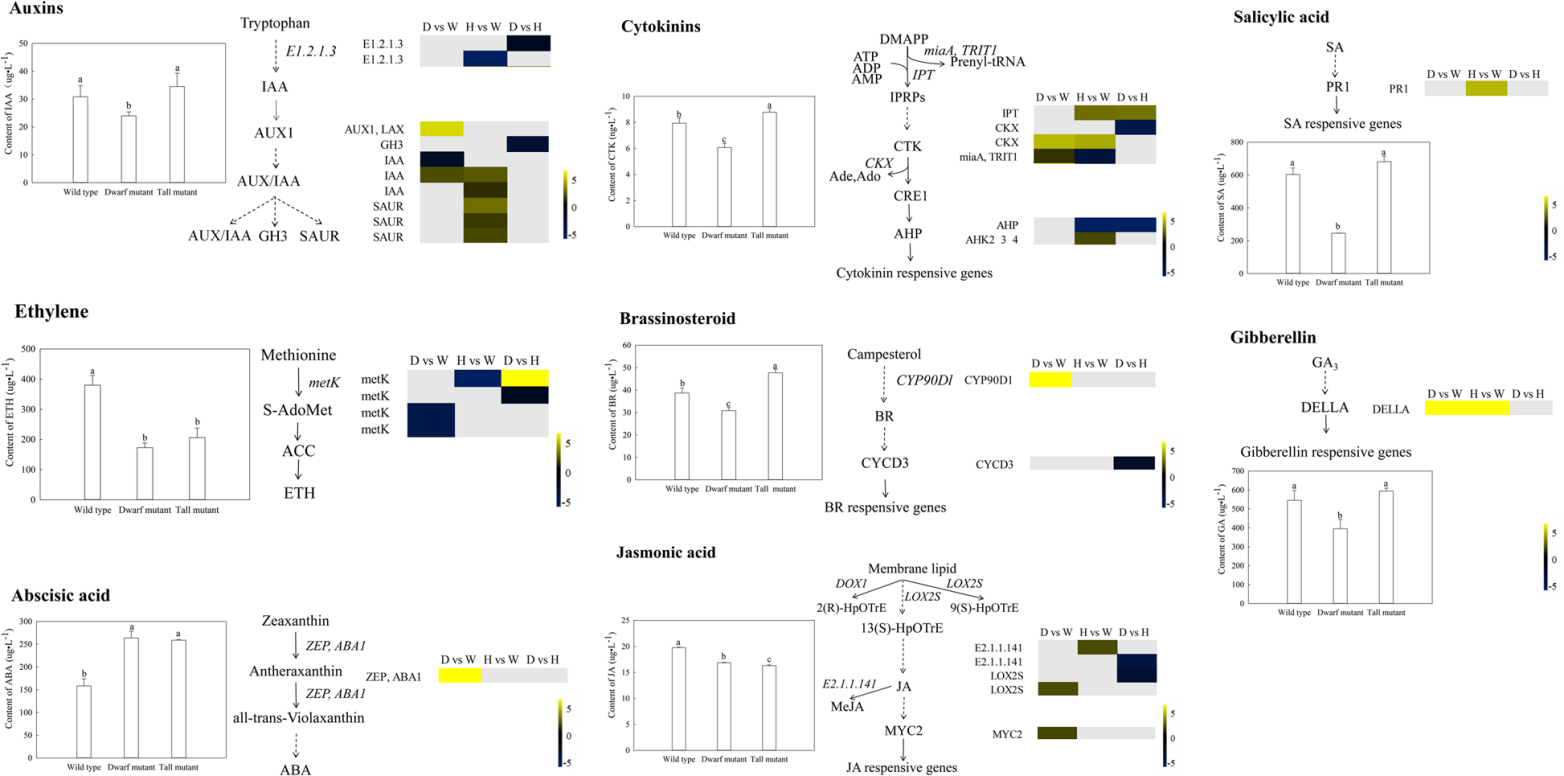
Heatmaps showing the relative changes in the expression of biosynthesis- and signal pathway-related genes in the *S. davidii* dwarf mutant, wild type and tall mutant. Yellow and blue indicate up- and downregulated transcripts, respectively, identified in the three comparisons, and white indicates no significant difference in expression. All genes are listed in Supplemental Table S4

DEGs in the *S. davidii* dwarf mutant, wild type and tall mutant annotated as being involved in known phytohormone biosynthesis and signaling pathways are shown in Fig. 4 and Supplemental Table S4. In the IAA biosynthesis pathway, the expression of the aldehyde dehydrogenase gene *E1.2.1.3* was significantly downregulated in the tall vs. wild-type and dwarf vs. tall groups. One *GH3*, one *AUX/IAA*, three *IAA*, and three *SAUR* genes showed significantly different expression profiles in the *S. davidii* dwarf mutant, wild type and tall mutant. One *IAA* gene (Cluster-12007.38985) was significantly upregulated in the dwarf mutant and tall vs. wild-type groups. In the CTK biosynthesis pathway, the cytokinin synthase gene *IPT*, which is involved in zeatin biosynthesis, was significantly upregulated in the tall vs. wild-type and dwarf vs. tall groups. Additionally, the CTK dehydrogenase gene *CKX* (Cluster-12007.675), which is involved in zeatin biosynthesis, was significantly upregulated in the dwarf and tall vs. wild-type groupss. One *AHP* and one *AHK2_3_4* gene showed significantly different expression levels in the *S. davidii* dwarf mutant, wild type and tall mutant, with the histidine-containing phosphotransfer protein gene *AHP* significantly downregulated in the tall vs. wild-type and dwarf vs. tall groups. In the GA biosynthesis pathway, the expression of the DELLA protein was significantly upregulated in the dwarf and tall vs. wild-type groups. In the BR biosynthesis pathway, the expression of the *CYP90D1* gene was significantly upregulated in the dwarf vs. wild-type groups, and one *CYCD3* gene was significantly downregulated in the dwarf vs. tall groups. In the jasmonic acid (JA) biosynthesis pathway, two *E2.1.1.141* and two *LOX2S* genes involved in α-linolenic acid metabolism showed significantly different expression profiles in the *S. davidii* dwarf mutant, wild type and tall mutant, and one *MYC2* gene was significantly upregulated in the dwarf vs. wild-type groups. In the SA biosynthesis pathway, the pathogenesis-related protein 1 gene *PR1* was significantly upregulated in the tall vs. wild-type groups. In the abscisic acid and ethylene biosynthesis pathways, carotenoid biosynthesis and cysteine and methionine metabolism genes showed significantly different expression levels in the *S. davidii* dwarf mutant, wild type and tall mutant.

### DEGs involved in flavonoid biosynthesis and phenylpropanoid biosynthesis

**Fig. 5:**
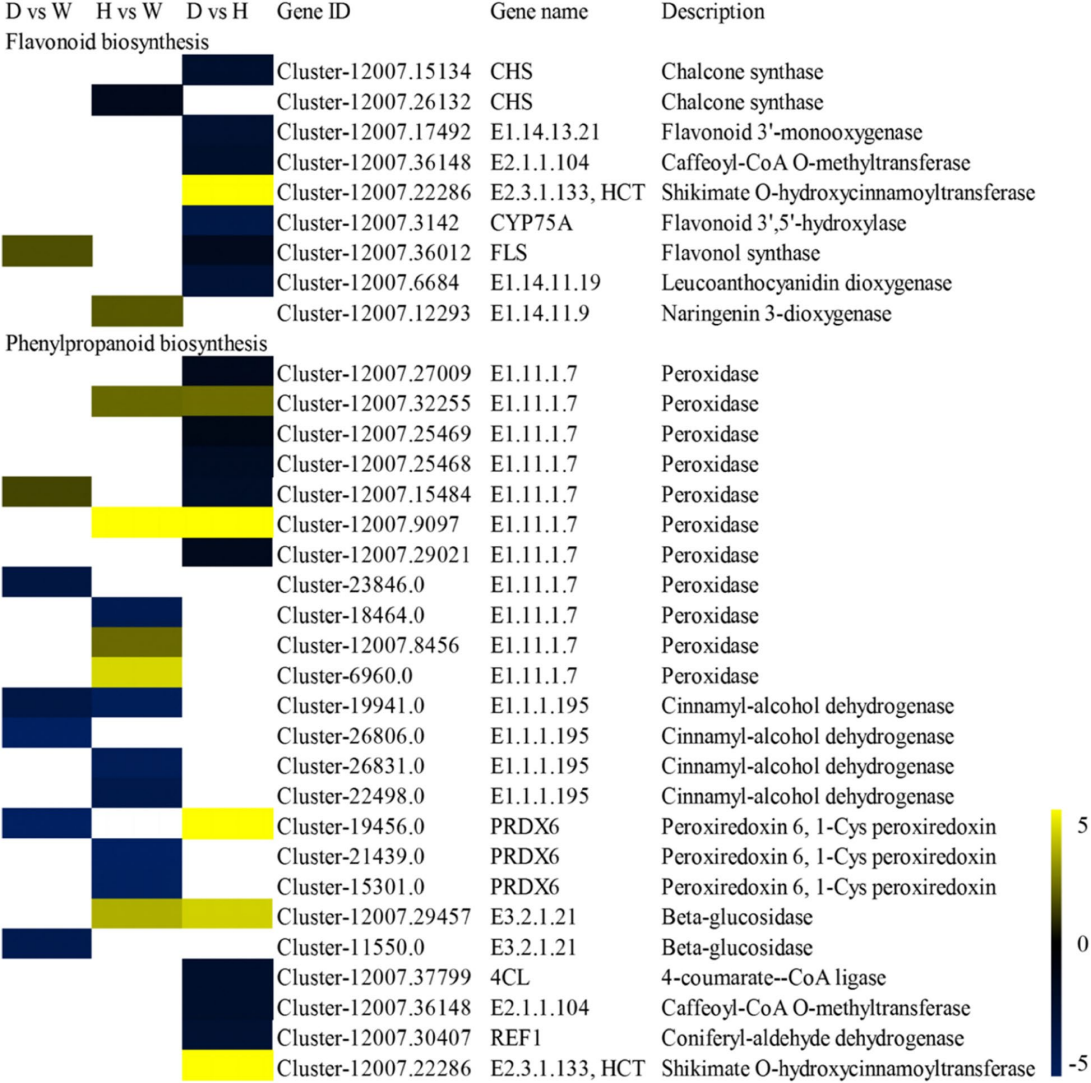
Heatmaps showing the relative changes in the expression of flavonoid biosynthesis- and phenylpropanoid biosynthesis-related genes of the *S. davidii* dwarf mutant, wild type and tall mutant. Yellow and blue indicate up- and downregulated transcripts, respectively, from the five comparisons, and white indicates no significant difference in expression. All genes are listed in Supplemental Table S5

Many secondary-metabolism genes were downregulated in the *S. davidii* wild type, dwarf mutant, and tall mutant. Regarding flavonoid biosynthesis, only the shikimate O-hydroxycinnamoyltransferase gene *E2.3.1.133* (*HCT)*, flavonol synthase gene *FLS*, and naringenin 3-dioxygenase gene *E1.14.11.9* were significantly upregulated in the dwarf vs. tall and dwarf and tall vs. wild-type groups. Regarding phenylpropanoid biosynthesis, the *E1.11.1.7* (Cluster-12007.32255, Cluster-12007.9097, Cluster-12007.8456, Cluster-6960.0), *E3.2.1.21* (Cluster-12007.29457), and *E2.3.1.133 (HCT)* (Cluster-12007.22286) genes were significantly upregulated in the *S. davidii* dwarf mutant, wild type and tall mutant (Fig. 5 and Supplemental Table S5)*.*

### Endogenous hormone concentrations

To better understand the involvement of endogenous hormones in plant height in the *S. davidii* wild-type, dwarf mutant and tall mutant, we quantified the concentrations of eight endogenous hormones, IAA, cytokinin (CTK), ethylene (ETH), abscisic acid (ABA), GA, BR, JA and salicylic acid (SA), in shoot apexes and lateral branch buds of the three genotypes using ELISA (Fig. 4). The results showed that the IAA, CTK, GA, BR and SA contents in the dwarf mutant were significantly lower than those in the wild type and tall mutant. ETH and JA contents in the dwarf mutant were significantly lower than those in the wild type. The ABA contents in the dwarf and tall mutants were significantly higher than that in the wild type*.*

### Validation of DEGs using qRT–PCR

**Fig. 6:**
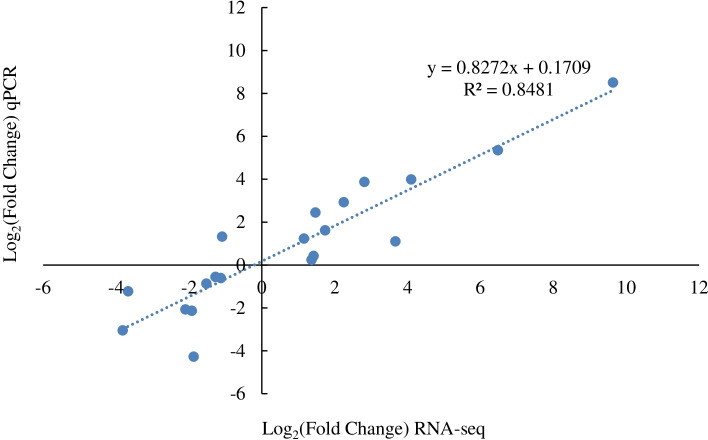
Correlation analysis of gene expression patterns determined by RNA-Seq and qRT–PCR

We randomly selected 20 DEGs for validation by qRT–PCR, including 4 genes from the dwarf vs. wild-type groups and 8 genes from the tall vs. wild-type and dwarf vs. tall groups. The input data were the log_2_(fold change (FC)) values of each gene determined by the two methods. The tested genes displayed the same expression patterns according to RNA-Seq or qPCR, and the R^2^ was 0.8481 (Fig. 6), indicating that the relative expression FCs between the two methods showed a high correlation.

### Detection of differential metabolites in *S. davidii*

Twelve samples were clearly distinguishable by principal component analysis (PCA), with the most effective variables being included in PC1 (30.57%) and PC2 (18.76%) (Fig. S1). The results showed good reproducibility and representativeness. To identify differences in secondary metabolite concentrations between the *S. davidii* dwarf and tall mutants, we performed liquid chromatography–high-resolution accurate tandem mass spectrometry (LC–HRMS–MS) analysis of shoot apexes from the *S. davidii* dwarf and tall mutants. The LC–HRMS–MS workflow produced a metabolite data table containing intensity values for 464 metabolites. The variable importance in projection (VIP) value of the first principal component of the partial least-squares discriminant analysis (PLS–DA) model was used, the FC was calculated as the ratio of the average value of each metabolite in the comparison group of all biological replicates, and these results were combined with the P value of the T test to identify differentially expressed metabolites. The thresholds were set to a VIP > 1.0, a multiple of difference FC > 1.2 or ROC < 0.833 and a *P* value < 0.05. According to these criteria, 56 significantly different metabolites were screened between the dwarf and tall mutants, among which 31 were downregulated and 26 were upregulated (Supplemental Table S6).

The significantly different metabolites were classified and annotated in LIPID MAPS, and a total of 8 significantly different metabolites were annotated in both the dwarf and tall mutants. The majority of the compounds were flavonoids, including glabrene, hesperidin, kaempferol, diosmetin 7-neohesperidoside, poncirin and rottlerin (Table 2). The concentrations of the fatty amide capsaicin and the steroid conjugate tauroursodeoxycholic acid were 0.25- and 2.28-fold higher in the tall mutant than in the dwarf mutant.

**Table 2:** Results of LC–HRMS–MS analysis of shoot apexes of the *S. davidii* dwarf and tall mutants

Annotation	Type	RT [min]	FC D/H	log2FC	*P* value	ROC	VIP	Up/Down
Capsaicin	Fatty amides	10.549	0.25	-2.00	0.0086	0.94	2.45	down
Glabrene	Flavonoids	12.16	2.48	1.31	0.0341	0.83	1.19	up
Hesperidin	Flavonoids	9.46	3.21	1.682	0.0012	0.92	1.67	up
Kaempferol	Flavonoids	9.841	0.25	-2.03	0.0053	0.94	1.98	down
Diosmetin 7-neohesperidoside	Flavonoids	8.988	3.26	1.70	0.0064	0.92	1.60	up
Poncirin	Flavonoids	12.072	2.32	1.21	0.0192	0.92	1.30	up
Rottlerin	Flavonoids	9.798	2.39	1.26	0.0180	0.89	1.25	up
Tauroursodeoxycholic acid	Steroid conjugates	13.539	2.28	1.19	0.0020	1.00	1.29	up

Abbreviations: *LC–HRMS–MS* liquid chromatography–high-resolution accurate tandem mass spectrometry, *RT* [min] retention time, *FC D/H* comparison of the multiple of the difference, *ROC* receiver operating characteristic curve area, *VIP* variable importance projection

The significantly differential metabolites were analyzed by Kyoto Encyclopedia of Genes and Genomics (KEGG) pathway analysis, and a total of 8 significantly differential metabolites were matched to 14 pathways (Table 3). The metabolites chlorogenic acid, kaempferol and scopoletin were involved in phenylpropanoid biosynthesis and flavonoid biosynthesis, and their concentrations were 0.30-, 0.25- and 0.26-fold higher, respectively, in the tall mutant than in the dwarf mutant.

**Table 3:** KEGG pathway annotation information

Metabolite	RT [min]	FC D/H	*P* value	KEGG_pathway_annotation	Up/Down
Cytisine	2.08	0.41	0.0392	Tropane, piperidine and pyridine alkaloid biosynthesis; Biosynthesis of secondary metabolites	down
N-Acetylornithine	1.451	2.57	0.0086	Arginine biosynthesis; Metabolic pathways; Biosynthesis of secondary metabolites; 2-Oxocarboxylic acid metabolism; Biosynthesis of amino acids	up
Chlorogenic acid	7.347	0.30	0.0285	Phenylpropanoid biosynthesis; Flavonoid biosynthesis; Stilbenoid, diarylheptanoid and gingerol biosynthesis; Biosynthesis of secondary metabolites	down
Kaempferol	9.841	0.25	0.0053	Flavonoid biosynthesis; Flavone and flavonol biosynthesis; Metabolic pathways; Biosynthesis of secondary metabolites	down
Phosphoric Acid	1.64	2.30	0.0156	Oxidative phosphorylation; Photosynthesis; Metabolic pathways; ABC transporters	up
Capsaicin	10.549	0.25	0.0086	Phenylalanine metabolism; Metabolic pathways; Biosynthesis of secondary metabolites	down
Scopoletin	7.353	0.26	0.0213	Phenylpropanoid biosynthesis; Biosynthesis of secondary metabolites	down
Nicotinamide	1.419	0.63	0.0216	Nicotinate and nicotinamide metabolism; Metabolic pathways	down

## Discussion

### Cell wall biosynthesis and expansion play a major role in plant height

The development of plant-height mutants is usually related to changes in cellular processes, such as morphogenesis and division [24]. In this study, the cells of the dwarf mutant were smaller and more densely arranged, while those of the wild type and tall mutant were larger and loosely arranged. Additionally, the dwarf and tall mutants showed significant reductions in phloem area relative to the wild type. The establishment of cell size and cell shape is related to the progression of the cell cycle, including the biogenesis of new cell plates and the key steps in cell wall deposition and metabolism [21].

Plant height is determined by the extent of cell wall extension, and plants achieve control over cell growth [25]. The cell wall is a three-dimensional network composed of polysaccharides, proteins, and other components, which are formed by the interaction between cellulose and pectin [22]. Cellulose plays an important role in regulating plant cell volume and determining cell size [23]. The *CESA* gene family encodes the catalytic subunits of cellulose synthase and is responsible for the biosynthesis of cellulose in plant cell walls [26]. Compared with the wild type, four DEGs encoding *CESA* subunits were significantly downregulated in the tall mutant, and the results were in accordance with the tall mutant phenotype regarding phloem area, pulp area, xylem area, total cross-sectional area, pelvic diameter and branch number, which showed lower values than those in the dwarf mutant. These results indicate that the cell wall biosynthesis and elongation abilities of the tall-mutant stems were reduced.

EXPs are a type of protein in the cell wall of plants that is mainly involved in cell growth, elongation and various cell wall modifications. It can reduce the tension of the cell wall network and effectively relax the cell wall [27, 28]. The role of EXPs has been demonstrated in many plants; for example, relative to the wild type, the dwarf rice mutant (pex1) shows a higher lignin content in the stem and higher expression levels of lignin-related genes [29]. The overexpression of *OsEXP4* in transgenic rice resulted in a 12% increase in plant height [30]. In our study, the expansin-A8, expansin-A4, and expansin-A1 genes presented significantly lower expression levels in the dwarf mutant than in the tall mutant, whereas the putative expansin-B2 gene presented significantly higher expression levels in the tall mutant than in the wild type, indicating that these genes may participate in increasing plant height and branch length by enabling cell expansion. Studies have also shown that increasing the expression level of an *EXP* gene may not always increase plant height and may cause lateral cell expansion to increase the plant stem diameter [31]. Wang et al. [32] found that the overexpression of an *EXP* gene in dwarf shengyin bamboo significantly increased its fiber and stem diameter. These results are consistent with the observations that expansin-A8 was significantly upregulated in the *S. davidii* dwarf mutant, while expansin-A17 gene was significantly downregulated in the *S. davidii* tall mutant; that the stem diameter of the tall mutant (approximately 1.74 cm) was thicker than that of the dwarf mutant (approximately 1.31 cm); and that the primary, secondary, and tertiary branch lengths of the tall mutant were greater than those of the dwarf mutant and wild type. These results showed that increasing the expression level of an *EXP* gene may not always increase the *S. davidii* height but may lead to increases in the stem diameter and branch length.

*XTH* encodes a cell wall modification enzyme found in higher plant cells that catalyzes the hydrolysis or transfer of xyloglucan molecules, thereby changing the structure of the cell wall [27]. Growth-promoting phytohormones have also been found to induce *XTH* gene expression; for example, brassinosteroids can regulate the activity of the *XTH* enzyme and increase the ductility of the cell wall. When *Arabidopsis thaliana* is treated with BR, the expression of the *AtXTH22* and *AtXTH24* genes is significantly increased, thereby promoting the elongation of the *Arabidopsis* cell wall [33]. In *Arabidopsis* dwf1 plants, the expression levels of the *AtXTH22* and *AtXTH24* genes are greatly reduced [34]. In this study, the *XTH30* gene was downregulated in the tall vs. wild-type groups, and the *XTH31* and *XTH22* genes were upregulated in the dwarf vs. wild-type and dwarf vs. tall groups, indicating that these genes are involved in the lateral cell expansion of *S. davidii* to increase the stem diameter and branch number in the dwarf mutant and wild type. In addition, the *XTH33* gene was upregulated in the tall vs. wild-type groups, and the *XTHA* gene was downregulated in the dwarf vs. tall groups, indicating that these genes are involved in the rapid differentiation and expansion of stem cells to increase the *S. davidii* plant height.

### Importance of plant hormones in determining plant height

Plant hormones play an important role in plant morphogenesis, growth and metabolism [35]. Blocking plant hormone metabolism and signal transduction pathways is one of the mechanisms of plant dwarfing. Studies have shown that plant dwarf mutations are closely related to plant hormones, including GA, BR and IAA [36].

Auxin and CTK act antagonistically to regulate root and shoot growth, the outgrowth of axillary meristems and their own synthesis and transport [37]. Auxin plays a regulatory role in all stages and levels of plant growth and development [38]. A previous study found that in the *CYP79b2 CYP79b3* double mutant (cytochrome P450 silenced), the hypocotyls of seedlings are shortened, the plants are dwarfed, and the content of auxin is reduced [39]. In this study, the aldehyde dehydrogenase gene in the IAA biosynthesis pathway was found to be significantly downregulated in the dwarf vs. tall and tall vs. wild-type groups, which may lead to reduced IAA synthesis in the dwarf and tall mutants, affecting dwarf mutant normal growth and resulting in a decrease in the branch number. *AUX/IAA, GH3* and SAUR are three major auxin response gene families [40]. The abnormal expression of key genes for auxin transport can cause plant dwarfing. In this study, the *GH3* (Cluster-18804.0) gene was observed to be significantly downregulated in the dwarf mutant relative to the tall mutant, indicating a reduced auxin response. The expression of *AUX1* and *IAA* was significantly upregulated in the dwarf and tall vs. wild-type groups. *AUX/IAA* is an auxin effector protein that plays a negative regulatory role in the dwarf mutant. When the auxin content increases in tall mutants, the transcriptional activators of auxin response ARFs are separated and bound to auxin, thereby regulating the expression of auxin response genes [4]. The positive regulatory *SAUR* gene may be involved in cell proliferation and cell diffusion in *S. davidii*, and this gene is upregulated in tall mutants. All of the results showed that these three genes affect plant height during *S. davidii* growth and development. The *CKX* function of the *CTK* biosynthesis pathway in plant type regulation has been reported. In rice, low *CKX* expression will cause the accumulation of *CK* in the inflorescence meristem, which will lead to an increase in rice tillers [37]. In our study, *CKX* (Cluster-12007.675) was upregulated in the dwarf and tall vs. wild-type groups but downregulated in the dwarf vs. tall groups (Cluster-12007.43915), which may cause *CTK* accumulation in the lateral branches of *S. davidii*, leading to an increase in branch number and a decrease in plant height in the *S. davidii* dwarf mutant. The cytokinin signal transduction gene AHP (Cluster-12007.48311) was downregulated in the tall vs. wild type and dwarf vs. tall groups, inhibiting its dominance in the *S. davidii* wild type and tall mutant and promoting increased branch height.

When plant GA synthesis is defective or insensitive to gibberellin, it will lead to a dwarf phenotype. The plant hormone GA, the receptor *GID1* and the inhibitor *DELLA* together constitute the GA signal transduction pathway [41]. The *DELLA* gene encodes a negative regulatory protein of GA signal transduction. If the *DELLA* functional region is destroyed, gibberellin cannot eliminate the effect of the encoded product on plant growth inhibition, resulting in plant dwarfing [42]. In this study, the GA content in the *S. davidii* dwarf mutant was significantly lower than those in the wild type and the tall mutant, and the *DELLA* gene was significantly upregulated in the *S. davidii* dwarf mutant. This may have occurred because when the GA content is low, the *GID1* receptor does not bind to GA, and the *DELLA* protein binds to the downstream target gene to inhibit its transcription, which results in the suppression of the *S. davidii* plant height and cell growth. The *DELLA* gene was significantly upregulated in the *S. davidii* tall mutant. This may have been due to the participation of the ubiquitin E3 ligase complex by adding a polyubiquitin chain to the *DELLA* protein to induce the degradation of the 26S protease complex pathway, thereby relieving the inhibitory effect of *DELLA* on plant growth [43].

Studies have shown that the deletion or mutation of genes, transcription factors, and enzymes related to BR synthesis and signal transduction pathways affects the elongation of plant cells, causing plants to exhibit a dwarfed phenotype [44]. For example, the rice BR-insensitive mutant d61 exhibits extreme dwarfing [45]. *CYP90D1* belongs to the cytochrome P450 (*CYP450*) family of genes and contains a conserved heme-binding domain. These enzymes catalyze the hydroxylation step of C23 in the BR synthesis pathway together. *CYP90D1* can positively regulate plant growth, including stem growth, hypocotyl elongation and petiole growth [46]. In this study, increased expression of the *CYP90D1* gene was detected in the dwarf vs. wild-type groups, indicating that its expression in the *S. davidii* dwarf mutant is positively regulated by the BR signal transduction pathway to increase branch number. *CYCD3* is a D-type plant cyclin gene that can promote the division of plant cells. The *CYCD3* gene is activated by CTK to promote cell division. *Arabidopsis* shows marked dwarfing when *CYCD3* is inhibited [47]. In this study, we found that *CYCD3* (cluster-12007.20005), which is involved in BR signal transduction, was significantly downregulated and that the BR content was significantly decreased in the dwarf vs. tall groups of *S. davidii*, which indicates that *S. davidii CYCD3* may be involved in the regulation of plant height and play a negative role in BR signal transduction, affecting the division of plant cells.

Two other endogenous inhibitory hormones exist—JA and SA. Many studies have shown that these hormones are also involved in plant development [48]. In this study, the expression of *MYC2* and *PR1* genes was shown to be significantly upregulated in dwarf and tall vs. wild-type groups, which is consistent with findings in bamboo, indicating that JA and SA probably inhibit plant height and branch number in the *S. davidii* dwarf mutant, but further research on this subject is needed [32]. Plant height is related to the balance among multiple hormones. Previous studies have shown that hormonal signals such as IAA, GA and BR transcription factors interact with each other to promote cell elongation in internode tissue [49, 50] and act as regulators of stem elongation [51]. For example, BR can promote the transport of IAA, IAA can promote the expression of the BR synthesis protein DWF4, and both IAA and BR signals can promote GA biosynthesis [52]. CTK and IAA act antagonistically to regulate plant height establishment [53]. In this study, auxin was shown to promote *S. davidii* dwarfing through a signaling pathway and by inhibiting the binding of the DELLA protein to downstream target genes. Auxin and CTK may also regulate *S. davidii* dwarfing through antagonism. Plant height control is complex and is influenced not only by plant hormones but also by environmental factors such as light, photoperiod, carbon sinks and nutrients. Based on our analysis of the data, we assume that the complex regulation of various signaling pathways involved in the coordination of cell wall biosynthesis and expansion affects cell and plant height. For example, *AUX1*, *DELLA*, and *MYC2* jointly regulate a series of genes, and these genes jointly regulate the *S. davidii* branch number. These genes are affected by IAA-, GA- and JA-coregulated proteins and consist mainly of cell wall synthesis and cell wall relaxation proteins, such as *EXP*s and *XTH*s, which regulate cell division.

### Importance of flavonoids in determining plant height

Phenylpropanes and flavonoid metabolites play an important role in plant growth and development. The secondary metabolic pathways of plant phenylpropanes affect many important plant traits, such as the synthesis of plant lignin, through the synthesis of various secondary metabolites, including flavonoids [54, 55]. This study screened 24 DEGs associated with phenylpropanoid biosynthesis, such as genes encoding peroxidase *(E1.11.1.7)*, cinnamyl-alcohol dehydrogenase *(E1.1.1. 195)*, peroxiredoxin 6,1-Cys peroxiredoxin *(PRDX6)*, beta-glucosidase *(E3.2.1.21)*, 4-coumarate–CoA ligase *(4CL)* and caffeoyl-CoA O-methyltransferase *(E2.1.1.104)*. The plant-specific peroxidase is Class. III peroxidase *(EC1.11.1.7)*, which is a gene downstream of the phenylpropane biosynthetic pathway that affects cotton fiber elongation and branch and root development [56, 57]. Eleven EC: 1.11.1.7 genes were detected in S. davidii, most of which were upregulated in the tall vs. wild-type groups, while others were downregulated in the dwarf vs. tall groups. In dwarf shengin bamboo, all peroxidase genes are upregulated, and the lignin content is increased [32], indicating that enough lignin is present to promote cell elongation and growth. This process increases the height of the *S. davidii* tall mutant and, conversely, reduces the height of the *S. davidii* dwarf mutant. Flavonoids control auxin transport, seed germination, prevention of oxidation of unsaturated fatty acids and scavenging of free radicals in plants [58, 59]. We found that the flavonoid biosynthetic pathway involves a series of chalcone synthase (CHS), flavonoid 3',5'-hydroxylase (F3′5'H) and flavonoid synthase (FLS) genes. In our study, FLS genes were upregulated in the dwarf vs. wild-type groups, which was consistent with the upregulation of FLS genes in dwarf rootstocks [60], indicating that FLS gene upregulation increased flavonoids in dwarf white spurge flowers, thereby reducing growth hormone transport and the dwarf phenotype. In contrast, FLS genes were downregulated in the dwarf vs. tall group, suggesting that FLS genes are associated not only with plant height but possibly also with seed morphology.

Metabolomics responds well to morphogenetic differentiation through differential metabolite differences and up- and downregulation relationships [61]. In this study, the tall mutant differed more markedly than the dwarf mutant compared to wild-type S. davidii; therefore, the tall mutant was used as a control, and the dwarf mutant was used for metabolomic analysis. A total of 56 differential metabolites were detected by LC–MS. These differential metabolites were related to the elongation of the stem and further dwarfing. The differential metabolites were classified and annotated in LIPID MAPS. Among them, the dwarf and tall mutants showed a total of 8 different metabolites, 6 of which were flavonoids (glabrene, hesperidin, kaempferol, diosmetin 7-neohesperidoside, poncirin and rottlerin), indicating that these metabolites play a role in the growth and development of *S. davidii*, especially in plant height morphogenesis. He et al. [62] showed that relative to wild-type Kentucky bluegrass, the plant height of the Kentucky bluegrass space mutant A16 was reduced, and the flavonoid content was significantly increased. A large number of studies have also shown that flavonoids can inhibit auxin transport and reduce plant height [63]. Our study found an increase in flavonoid content in the dwarf mutant compared to the tall mutant, which implied that the difference in the auxin content of the *S. davidii* dwarf mutant may be caused by differences in the auxin metabolic pathway or the alteration of flavonoid contents in the dwarf mutant of *S. davidii*. This difference may also be caused by variations in auxin within the plant, and the specific mechanisms involved need to be further studied. Through KEGG analysis, we found that three metabolites, chlorogenic acid, kaempferol and scopoletin, were involved in phenylpropanoid biosynthesis and flavonoid biosynthesis, all of which were downregulated. This finding may be related to the color or morphology of *S. davidii* seeds. Research by He et al. [62] showed that A16 seeds are browner than WT seeds, and the main color produced by flavonoids in the plant chromatogram ranges from light yellow to blue–purple [64]. The results of this experiment showed that the contents of chlorogenic acid, kaempferol and scopoletin in the dwarf mutant were significantly higher than those in the tall mutant; therefore, the yellow or light-yellow seeds of the *S. davidii* dwarf mutant may be caused by decreases in the contents of these three metabolites. Therefore, flavonoids can not only regulate the transport and metabolism of auxin but also integrate signaling pathways such as those of auxin and other hormones and transcriptional regulation to regulate the development of plant height.

## Conclusions

Phenotypic and anatomical observations found that the dwarf mutant displayed a significant decrease in plant height, while the tall mutant displayed a significant increase in plant height compared to the wild type. The cells of the dwarf mutant were smaller and more densely arranged, while those of the wild type and the tall stem type were larger and loosely arranged. Transcriptomic analysis of dwarf *S. davidii* identified a number of differentially expressed genes involved in cell wall biosynthesis, expansion, phytohormone biosynthesis, signal transduction pathways, flavonoid biosynthesis and phenylpropanoid biosynthesis. Through the metabolomic analysis of dwarf *S. davidii*, 8 significantly different flavonoid compounds were annotated to LIPID MAPS. KEGG analysis showed that three metabolites, chlorogenic acid, kaempferol and scopoletin, were involved in phenylpropanoid biosynthesis and flavonoid biosynthesis. These findings provide important information for use in determining the molecular genetic control of *S. davidii* plant height.

## Materials and methods

### Selection of dwarf mutants

Dwarf mutant and tall mutant materials were obtained through physical mutagenesis by ^60^Co-γ ray treatment (mutagenic dose of 120 KR). The wild type was used as a control in all experiments. All materials were sown and grown in a field in Guiyang, China, under similar conditions. The endogenous hormone content was measured at the mature stage in June 2019. The surfaces of the leaves and stems of the topmost internodes were also subjected to histological observations.

### Sample collection for RNA-seq and transcriptome sequencing

To minimize the transcriptional variation introduced by changes in the environment, *S. davidii* shoot apexes and lateral branch buds were collected from wild-type (W), dwarf mutant (D) and tall mutant (H) plants at the mature stage with three biological replicates. These samples were named W1, W2, W3, D1, D2, D3, H1, H2 and H3.

The total amount of RNA employed as the input material for RNA sample preparation was 1.5 μg per sample. Following the manufacturer's instructions, sequencing libraries were created using the NEBNext UltraTM RNA Library Prep Kit for Illumina (NEB, USA), and index codes were added to assign the sequences to each sample.

Using poly-T oligo-attached magnetic beads, mRNA was extracted from total RNA. In NEBNext First Strand Synthesis Reaction Buffer (5X), fragmentation was carried out utilizing divalent cations at high temperatures. M-MuLV Reverse Transcriptase and random hexamer primers were used to create first-strand cDNA (RNase H-). Next, second-strand cDNA synthesis was carried out utilizing DNA Polymerase I and RNase H. Exonuclease/polymerase activities were used to convert the remaining overhangs into blunt ends. NEBNext adaptors with hairpin loop topologies were ligated after adenylation of the 3' ends of DNA fragments in preparation for hybridization. The library fragments were purified using the AMPure XP system (Beckman Coulter, Beverly, USA) to preferentially pick cDNA segments 250,300 bp in length. Then, 3 μl of USER Enzyme (NEB, USA) was added to size-selected, adaptor-ligated cDNA, followed by 15 min at 37 °C and 5 min at 95 °C before PCR. Next, PCR was carried out using Phusion High-Fidelity DNA Polymerase, universal PCR primers, and an Index (X) Primer. Next, the PCR products were purified (AMPure XP system), and the library quality was evaluated using an Agilent Bioanalyzer 2100 system. Finally, qualified cDNA libraries were sequenced using the Illumina HiSeqTM 2000 platform (BGItech, Shenzhen, China).

### Clean read alignment of the dwarf mutant reference genome and transcriptomic analysis

The clean reads were aligned to the *S. davidii* sequence data. The reads were mapped to a reference genome using BWA software, and the reference genes were mapped using Bowtie software [65]. Integrative Genomics Viewer (IGV) was used to visualize the alignment findings. These two software packages were used to compute the genome alignment rate and gene mapping rates (the ratio of reads that were mapped to a matching gene in the gene annotation file). After calculating the alignment results for quality control, the expression level of each gene was evaluated by RNA-Seq using the Expectation Maximization (RSEM) method [66] in fragments per kilobase per million reads (FPKM). This approach can prevent the effects of diverse cDNA library transcript lengths and sequencing depths.

### Identification of DEGs and functional analysis

All DEGs were submitted to GO term annotation (http://www.geneontology.org/) [67] and KEGG pathway enrichment (http://www.genome.jp/kegg/) [68] studies for functional analysis. When the P value after Bonferroni adjustment (Q value) was ≤ 0.05, the GO word and KEGG pathway enrichment results were judged significant. WEGO software was also utilized to conduct statistical analyses of GO functional categories and KEGG pathway enrichment.

### Validation of quantitative real-time PCR

We employed qRT–PCR to confirm the expression levels of 20 selected genes. High-throughput sequencing was performed on RNA samples, and reverse transcription was performed with a HiFiScript cDNA Synthesis Kit (Cwbiotech). The gene-specific primers were created with the PerlPrimer program and are reported in Supplementary Table S7. Using an Applied Biosystems CFX ConnectTM Real-Time System and UltraSYBR Mixture, we performed qRT–PCR (Cwbiotech). The following thermal cycling parameters were used: 95 °C for 10 min, followed by 40 cycles of 95 °C for 15 s and 60 °C for 1 min in a 20 μl volume. Each reaction consisted of three biological replications, with the actin gene serving as an internal reference gene. The 2-△△Ct method [69] was used to compute the relative expression level of each gene in the dwarf vs. wild-type, tall vs. wild-type, and dwarf vs. tall groups.

### Secondary metabolite analysis

A commercial service provider performed broad-targeted metabolomic profiling (Novogene, Beijing, China). The D and H samples were analyzed using LC–MS/MS. Six biological replicates were carried out, with each sample run in triplicate. The LC–MS/MS studies were carried out at Novogene Co., Ltd. (Beijing, China) utilizing a Vanquish ultrahigh-performance liquid chromatography (UHPLC) system paired with an Orbitrap Q ExactiveTM HF-X mass spectrometer (Thermo Fisher, Germany).

The samples were injected into a Hypesil Gold column (100 × 2.1 mm, 1.9 μm) with a 17-min linear gradient and a flow rate of 0.2 mL/min. In positive polarity mode, the eluents were eluent A (0.1% FA in water) and eluent B. (methanol). Eluent A (5 mM ammonium acetate, pH 9.0) and Eluent B (5 mM ammonium acetate, pH 9.0) were used in negative polarity mode (methanol). The solvent gradient was established as follows: 2% B, 1.5 min; 2–100% B, 12.0 min; 100% B, 14.0 min; 100–2% B, 14.1 min; and 2% B, 17 min. The Q ExactiveTM HF-X mass spectrometer was used in positive/negative polarity mode, with a spray voltage of 3.2 kV, a capillary temperature of 320 °C, a sheath gas flow rate of 40 arb, and an aux gas flow rate of 40 arb [70].

Compound Discoverer 3.1 (CD3.1, Thermo Fisher) was used to conduct peak alignment, peak selection, and quantification for each metabolite from the raw data files obtained by UHPLC–MS/MS. The following settings were set: retention time tolerance of 0.2 min; real mass tolerance of 5 ppm; signal intensity tolerance of 30%; signal/noise ratio of 3; and minimum intensity of 100,000. The peak intensities were then normalized to the overall spectral intensity. Based on additive ions, molecular ion peaks, and fragment ions, the normalized data were utilized to determine the molecular formula. The peaks were then matched with the mzCloud (https://www.mzcloud.org/), mzVault, and MassList databases to provide precise qualitative and quantitative findings. The statistical tools R (R version R-3.4.3), Python (Python 2.7.6 version), and CentOS were used to conduct the analysis (CentOS release 6.6). When the data were not normally distributed, the area normalization approach was used to try normal transformations.

### Semithin sections and light microscopy for histologic observations

Specimen slices were obtained by paraffin sectioning, which is the most widely used conventional sectioning technique [32]. Stems and leaves of W, D and H plants at the mature stage were first fixed in FAA (stems: 5, formalin: 5, acetic acid: 75%, alcohol 90% by volume; leaves: 5, formalin: 5, acetic acid: 50%, alcohol 90% by volume), and suction was applied with an aspirator pump overnight at 4 °C. The samples were rinsed with distilled water multiple times to wash off the fixative solution and were then dehydrated in a graded ethanol series (50, 70, 95 and 100%) before embedding in paraffin. After drying (approximately seven days at 37 °C), transverse sections were cut from the embedded blocks with a YD-1508R rotary slicer. The sections were stained with Safranin O-Fast Green (Servicebio G1031) for 30–660 s at 25 °C, placed in clean xylene for 5 min, sealed with neutral gum, and observed with a Nikon Eclipse 80i light microscope. All images were captured with a Nikon DS-RiL camera (Nikon, Japan). Finally, the images were analyzed with Image-Pro Plus 6.0 (Media Cybernetics, Inc., Rockville, MD, USA).

### Endogenous hormones content determination

The contents of IAA, CTK, ETH, ABA, GA, BR, SA and JA in the shoot apexes and lateral branch buds of the *S. davidii* dwarf mutant, wild type and tall mutant were determined using the corresponding Solarbio detection kits (Beijing Solarbio Science & Technology Co., Ltd., Beijing).

### Statistical analysis

All plant height, crown diameter, stem diameter, internode number, branch length, leaf index, seed morphology and other data were collected in MS Excel 2007. The statistical analysis of all differences between the means was performed by using ANONA and DUNCAN with the IBM SPSS statistics 20 program. Image-Pro Plus 6.0 software was used for data analysis of the slices, and Origin 2018 was used for mapping.

## Supplementary Information

**Additional file 1:** **Fig. S1.** Principal component analysis (PCA) of metabolites

**Additional file 2:** **Table S1.** DEGs in D vs. W, H vs. W and H vs. D.

**Additional file 3:** **Table S2.** GO enrichment analysis of differentially expressed genes of D vs. W, H vs. W and H vs. D of Sophora davidii.

**Additional file 4:** **Table S3.** Relative changes in the expression of lignin biosynthesis-related genes.

**Additional file 5:** **Table S4.** DEGs involved in phythormone biosynthesis and signal transduction pathways.

**Additional file 6:** **Table S5.** DEGs involved in flavonoid biosynthesis and phenylpropanoid biosynthesis.

**Additional file 7:** **Table S6.** Fifty-seven significantly different metabolites in both the dwarf and tall mutants.

**Additional file 8:** **Table S7.** Gene-specific primers used in gene expression analysis by qPCR.

## Data Availability

All data supporting the findings were contained in the manuscript and its supplementary files except the RNA-seq raw data. And all the RNA-seq raw data were uploaded in the SRA of NCBI (PRJNA783425).
