# Supplementary figures and images for: Morphological, transcriptomic and metabolomic analyses of Sophora davidii mutants for plant height
Source: BMC Plant Biol. 2022 Mar 25;22:144. doi: 10.1186/s12870-022-03503-1 (PMC8951708; doi:10.1186/s12870-022-03503-1)

*
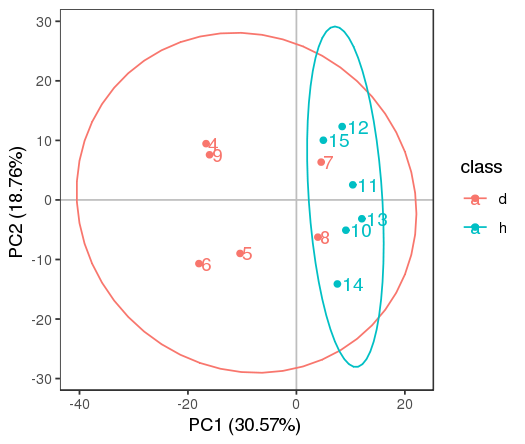
*

Figure S1. Principal component analysis (PCA) of metabolites.

Supplement: Supplementary file 1 — Additional file 1: Fig. S1. Principal component analysis (PCA) of metabolites [file 12870_2022_3503_MOESM1_ESM.docx]
